# Identity leadership and employees’ creative performance: a serial mediation model of relational energy and organizational embeddedness

**DOI:** 10.3389/fpsyg.2025.1581776

**Published:** 2025-11-20

**Authors:** Ki Baek Jung, Seung-Wan Kang, Suk Bong Choi

**Affiliations:** 1 Department of Business Administration, Korea National University of Transportation, Chungbuk, Republic of Korea; 2 College of Business, Gachon University, Seongnam, Republic of Korea; 3 College of Global Business, Korea University, Sejong, Republic of Korea

**Keywords:** identity leadership, relational energy, organizational embeddedness, creative performance, serial mediation

## Abstract

**Introduction:**

The aim of this study is to examine the relationship between identity leadership and creative performance. This study also examines the mediating roles of relational energy and organizational embeddedness in this causal relationship. Furthermore, a serial mediation model is applied, expecting that identity leadership will enhance the creative performance of organizational members through serial mediation paths.

**Methods:**

An empirical analysis was conducted with 397 employees working in Korean manufacturing and service firms. We conducted a confirmatory factor analysis to test result validity and used hierarchical regression to examine the direct and mediating effects. Additionally, we also used the Hayes (2013) Process Macro model to test a serial mediating effect.

**Results:**

The results showed that identity leadership was positively related to creative performance. Relational energy and organizational embeddedness mediated the relationship between identity leadership and creative performance. Last, identity leadership increased relational energy, enhancing organizational embeddedness and leading to higher creative performance through a serial mediation effect.

**Discussion:**

Our findings confirmed the role of leaders in enhancing the creative performance of employees. Furthermore, our empirical results revealed specific pathways through which identity leadership influences employee attitudes and performance. In doing so, we contribute to the body of research on leadership and social identity theory. Finally, we provide practical implications for managers who want to facilitate employee creative performance.

## 1 Introduction

Changing technology and business environments compel organizational leaders to pursue challenges and innovation (Gui et al., 2024; Wang et al., 2017). Leadership influences organizational performance by facilitating the efficient execution of tasks, fostering the generation of creative ideas, and encouraging innovative behaviors (Puni et al., 2022; Sarooghi et al., 2015; Zhang et al., 2022). Although the critical role of leadership is increasingly recognized, effective leadership styles vary across cultures (Den Hartog and De Hoogh, 2024). When comparing Eastern and Western cultures, the literature utilizes the concepts of collectivism and individualism to explain cultural characteristics (Oyserman et al., 2002). A collectivist culture emphasizes the relational or collective self-concept (Li and Cropanzano, 2009). Thus, employees in a collectivist culture have an attitude that allows individual sacrifices to achieve organizational goals and missions.

Traditionally, South Korea is characterized by low individualism and high in-group collectivism (Lyons et al., 2014). In collectivist cultures, organizations show high cohesion to achieve collective goals and strive to achieve organizational goals through a strong leadership style. As such, the traditional Korean leadership style encourages hard work and strict obedience from employees (Ulrich, 2019). However, recently in Korea, horizontal organizational structures are being designed and an organizational culture encouraging personal consideration is increasing. Moreover, the recent rapid changes in the business environment and increasing desire for personal growth among employees have intensified turnover. Thus, we argue that to respond to changes in the business environment and develop core competencies in South Korea, such as a collectivist culture, a unique leadership style is necessary. Korea’s culture emphasizes duty and loyalty to collective goals and the maintenance of group harmony (Lyons et al., 2014). Exploring effective leadership is especially crucial for Korean firms seeking to transform into more cohesive and collaborative organizations while achieving higher levels of creative performance. Therefore, we anticipate that a leadership style emphasizing group identity is essential in fostering employee alignment with and commitment to the organization. In this context, this study examines the impact of identity leadership and applies social identity theory to leadership practices.

Social cognition theory has been widely acknowledged as a prominent framework for explaining how individuals’ actions are shaped and affect performance within particular social environments (Bandura, 1986, 1997; Smith and Hitt, 2005). Bandura (1991) argued that individuals tend to model their behavior by observing and internalizing signals from significant others within their social environment. In this vein, we assumed that the effectiveness of identity leadership can be understood through a continuous cycle of social learning. For example, identity-based leaders function as behavioral models who embody the group’s shared values, norms, and goals. Through observational learning, followers cognitively and emotionally internalize these modeled behaviors from identity leadership, and begin to align their perceptions, motivations, and actions with the collective identity of the group. In addition, as followers observe their leader exemplifying the group’s identity, their sense of self-efficacy as well as the group’s shared collective efficacy can be enhanced. We believe that this social cognitive mechanism underlying identity leadership can be an effective theoretical lens for explaining the positive impact of identity leadership on employees’ creative performance by promoting cognitive alignment among followers on group identity and improving self- and collective-efficacy beliefs and creative-oriented behaviors.

In collective cultures in East Asia, identity and cooperation arise from maintaining relational harmony and promoting group cohesion (Brewer and Chen, 2007). Therefore, we expected positive effects from the identity leadership of Korean organizational leaders. In addition, we focused on intra-organizational relationships and the attractiveness that binds employees to the organization. Therefore, this study assumed that identity leadership would positively affect relational energy and organizational embeddedness. Employees gain energy through interactions with leaders and peers. Energy enhances the behavior and motivation of organizational members and affects job performance (Owens et al., 2016). Specifically, relational energy is influenced by leadership. A higher level of relational energy is obtained through positive interactions with leaders (Yang et al., 2019; Atwater and Carmeli, 2009). Reportedly, this relational energy positively affects job performance, work effort, organizational citizenship behavior, and creativity (Mao et al., 2022; Özkan et al., 2023; Yang et al., 2019). Thus, relational energy within the organization is a key antecedent of individual effort and work behavior. However, prior research has not focused on the role of relational energy in the relationship between leadership and creative performance. Therefore, this study aims to analyze this role.

This study also focuses on the role of organizational embeddedness in the relationship between leadership and creative performance. Embeddedness, based on Lewin’s (1951) field theory, explains the factors that prevent individuals from leaving the organization (Mitchell et al., 2001; Orie and Semeijn, 2022). Job embeddedness comprises fit, links, and sacrifice (Mitchell et al., 2001). Fit refers to the degree to which an individual perceives that their job and organization are compatible with their values and goals. Links refer to the extent of formal and informal connections with colleagues, teams, and the organization (Mitchell et al., 2001). Sacrifice refers to the perceived cost of physical and psychological benefits that would be lost if leaving the current organization (Mitchell et al., 2001). Embeddedness is influenced by factors including rewards, growth opportunities, procedural fairness, perceived organizational support, organizational commitment, and leader-member exchange (Chan et al., 2019; Nguyen et al., 2017; Harris et al., 2011). Organizational embeddedness reduces turnover intentions and turnover (Harris et al., 2011), while enhancing creative performance, innovative behavior, job satisfaction, affective commitment, and performance (Chan et al., 2019; Harris et al., 2011; Karatepe, 2016; Ng and Feldman, 2010b; Tian et al., 2016; Yang et al., 2019). Although organizational embeddedness is expected to influence employees’ attitudes and behaviors and determine organizational performance, limited research analyzes the impact of leadership on organizational embeddedness. Therefore, this study aims to explain the role of organizational embeddedness in the relationship between identity leadership and creative performance.

Previous studies identified the direct effects of identity leadership and focused on specific contexts (Stevens et al., 2021; Van Dick et al., 2018) including sports (Fransen et al., 2020) politics (Monzani et al., 2024) and education (Hung et al., 2025). However, efforts to apply it in business research have been insufficient. In addition, relationships with specific outcome variables such as organizational identification and well-being have been explored (Krug et al., 2020; Krug et al., 2021). However, prior studies are limited in explaining the effectiveness of identity leadership within firms. In particular, analysis of the processes through which identity leadership affects employee performance is lacking. Therefore, this study aims to explore the process through which identity leadership leads to creative performance. Here, a serial mediation model should be applied, as relational energy and organizational embeddedness are expected to serially influence this process. First, we explain the positive relationship between identity leadership and creative performance. Second, relational energy, which comprises positive emotions and moods derived from interpersonal interactions (Quinn and Dutton, 2005), is expected to mediate the relationship between identity leadership and creative performance. Third, organizational embeddedness, characterized by the desire to remain within the organization, is expected to mediate the relationship between identity leadership and creative performance. Last, this study aims to reveal a serial mediation process wherein identity leadership enhances relational energy, which positively influences organizational embeddedness, ultimately leading to increased creative performance.

## 2 Theoretical background and hypotheses

### 2.1 Identity leadership and creative performance

According to social identity theory, individuals perceive themselves as psychologically connected to organizations, which share characteristics with their own identity (Boezeman and Ellemers, 2007; Tajfel and Turner, 1979). Individual identity is formed as individuals internalize the attitudes, beliefs, values, emotional responses, and behavioral norms shared among employees of the same organization or society (Stets and Burke, 2000). Aligned with social identity theory, as individuals increasingly identify with the same identity as their organization, their behavior shifts from “me” to “we” in thinking and decision-making (Brewer, 1991; Turner et al., 1987). According to Brewer (1991) and Turner et al. (1987), individuals come to think and act in terms of “we” rather than “me” as they increasingly identify their own image and capabilities with those of the organization, viewing personal growth as synonymous with organizational growth. Ultimately, individuals are motivated to act in ways that benefit both themselves and the organization to maintain and enhance their self-esteem (Turner et al., 1987). Traditional leadership theories focused on leader characteristics or leader-follower relationships, whereas social identity theory has highlighted leadership styles emerging from group processes (Hogg, 2001; Tajfel and Turner, 1979). After this perspective emerged, leadership research applied social identity theory, focusing on leader identity prototypicality (Hogg, 2001). Prior studies emphasize the ongoing discussion on the importance of social identity in leadership (Reicher et al., 2005).

Identity leadership as a concept is based on social identity (Tajfel and Turner, 1979) and self-categorization theory (Turner et al., 1987). Its characteristics and components have recently been discussed. Identity leadership comprises four factors: identity prototypicality (being one of us), identity advancement (doing it for us), identity entrepreneurship (crafting a sense of us), and identity impresarioship (making us matter) (Steffens et al., 2014). First, identity prototypicality refers to leaders exemplifying organizational characteristics as model members, embodying the organization’s vision, and representing the team (Steffens et al., 2014). Second, identity advancement involves leaders’ actions to promote organizational benefits, remove hindrances, and contribute to goal achievement (Steffens et al., 2014). Third, identity entrepreneurship entails leaders internalizing a shared sense of “us” among employees, fostering cohesion among diverse backgrounds and characteristics. Last, identity impresarioship signals the importance of organizational existence and identity, enabling employees to sustain their roles and activities, preventing organizational division, and fostering continual manifestation of identity (Steffens et al., 2014). Previous research on identity leadership was conducted in various contexts including athletes (McLaren et al., 2021), in the public sector and corporate organizations (Mols et al., 2020; Steffens et al., 2020), and politics (Van Dick et al., 2019). Existing studies were both quantitative (Krug et al., 2021) and qualitative (Smith et al., 2018). These works found that identity leadership was positively related to team identification, trust, job satisfaction, and organizational citizenship behavior (Van Dick et al., 2018), and negatively impacted burnout (Van Dick et al., 2021).

This study expects that identity leadership positively impacts creative performance for the following reasons. Success in various task teams such as R&D, advertising, and production relies on new ideas and creative problem-solving. In this context, employees need motivation to contribute to team success through creative efforts. According to social identity theory, motivation can be facilitated through identity impresarioship, fostering individuals’ sense of belonging and identification with the team (Ashforth and Mael, 1989; Dutton et al., 1994; Mael and Ashforth, 1992; Van Dick, 2001; Van Knippenberg, 2000). Thus, identity leadership fosters a sense of unity among employees in the team. Consequently, employees are likely to engage in proactive problem-solving and generate new ideas for team success. Therefore, it is expected that employees’ creative efforts and thinking influence creative performance.

Furthermore, identity leadership integrates employees’ self-concept with team identity. When individuals’ identity aligns with team identity, employees can be motivated to overcome threats to the team’s status and goals (Hirst et al., 2009). Employees who perceive threats and image decline of the team also perceive these as affecting themselves. Therefore, employees may facilitate knowledge acquisition and strategies to contribute to creative performance to cope with external threats (Elliot and McGregor, 2001; Fisher and Ford, 1998; Hirst et al., 2009). Thus, employees strive for creative performance, organizational change, and innovation to ensure organizational survival, because they recognize that organizational harm can adversely affect individuals (Elliot and McGregor, 2001; Fisher and Ford, 1998; Hirst et al., 2009). Individuals may experience conflicts between their team member and personal roles, which can stem from conflicts between team and individual interests. Identity leadership aligns an employee’s identity with that of the team and organization. Specifically, identity leadership enhances cohesion within the organization and promotes the pursuit of team goals. Employees with a high level of team identity increase cooperation among colleagues (Van Vugt and De Cremer, 1999). Moreover, high levels of team identity prevent individual interests that hinder team benefits, thus enabling a focus on organizational goals (Ellemers et al., 2004). Therefore, identity leadership leads to constructive team processes and can positively impact creative outcomes. Thus, we hypothesized:

*Hypothesis 1* (H1). Identity leadership is positively related to creative performance.

### 2.2 Mediating effect of relational energy

Relational energy refers to a psychological boost derived from interpersonal interactions that enhances one’s capacity to perform work (Owens et al., 2016). Energy enhances employee motivation and focus on their tasks, enabling them to better achieve organizational goals (Quinn et al., 2012). In this context, relational energy is a psychological resource derived from interactions with leaders and colleagues, which enhances motivation and job performance. Previous studies confirmed leadership as an antecedent of relational energy, and relational energy positively impacts job engagement, job performance (Owens et al., 2016), work effort, and organizational citizenship behavior (Özkan et al., 2023). From a psychological perspective, energy has been a focus in Western cultures, necessitating an exploration of the relationship between leadership and performance in Eastern cultures (Yang et al., 2019). Therefore, this study expects that identity leadership that emphasizes “we” will be closely related to relational energy. Moreover, we intend to examine the mediating role of relational energy in the relationship between identity leadership and employee performance.

We argue that relational energy positively mediates the relationship between identity leadership and creative performance for the following reasons. Identity leadership combines individual and team identity, which creates team processes that help achieve the organization’s vision and goals (Steffens et al., 2014). In this process, leaders interact with members and internalize the “we” among employees. Since relational energy is a psychological resource obtained through interactions with leaders (Owens et al., 2016), employees’ observing and learning from the leader’s actions to establish identity enable them to gain relational energy through interactions. Identity leadership is developed based on social identity and self-categorization theory (Steffens et al., 2014). Self-categorization theory posits that individuals classify themselves and distinguish between their own organization and others through social categorization processes (Lau, 1989; Tajfel and Turner, 1986). When establishing identity, distinguishing between in-groups and out-groups enables employees with a team identity to develop strong cohesion with the team, leader, and colleagues (Hogg and Terry, 2000). This strong bond and communication with leaders can enhance relational energy.

Creative performance, which is rooted in employee creativity, can be achieved through new attempts that deviate from existing practices and procedures. Employees’ sustained effort in the creative process requires a high level of energy (Atwater and Carmeli, 2009). Relational energy is a resource necessary for creativity and creative problem-solving because it helps employees overcome challenges and difficulties in their tasks (Yang et al., 2021). Employees with high levels of energy can involve themselves in the creative process (Atwater and Carmeli, 2009; Mao et al., 2022). Creativity needed for creative performance requires diverse and new processes, underpinned by the efforts and persistence of organizational members. According to the conservation of resources theory, employees who gain psychological resources from the organization and leaders tend to reinvest these resources for the organization and team (Hobfoll, 1989, 2001). This suggests that employees who receive high levels of relational energy from their leaders can invest more time and energy into problem-solving processes, likely offering new and valuable solutions to the organization (Yang et al., 2021). Conversely, employees with low levels of relational energy may struggle to make sustained efforts for the organization, negatively impacting creative performance. In addition, relational energy can positively influence creativity by stimulating high levels of positive emotions (Baker, 2019; De Dreu et al., 2008; Owens et al., 2016). Thus, employees’ relational energy fosters positive emotions toward the organization and leaders, forming a positive attitude toward their tasks, which enhances creative performance.

Essentially, employees’ identity through identity leadership influences their attitudes and behaviors (Ashforth and Mael, 1989). Specifically, the concept of “we” can promote their relational energy. As an important resource, energy improves behavioral capability and motivation (Quinn et al., 2012). Relational energy stimulates positive emotions and influences positive attitudes. Therefore, employees with a high level of relational energy can promote creative performance based on employees’ positive psychological resources. Therefore, we hypothesized:

*Hypothesis 2* (H2). Relational energy positively mediates the relationship between identity leadership and creative performance.

### 2.3 Mediating effect of organizational embeddedness

Embeddedness refers to a state of being so closely integrated with or entangled in a certain entity that it becomes difficult to distinguish between them. This implies that an individual embedded in an entity has formed a strong, inseparable relationship. In previous studies, job embeddedness was used to explain why employees remain in an organization despite better opportunities elsewhere, emphasizing the process of maintaining employment relationships (Grewal and Slotegraaf, 2007; Halbesleben and Wheeler, 2008; Ng and Feldman, 2010a). Aligned with previous studies, this research uses the terms “job embeddedness” and “organizational embeddedness” interchangeably (Ng and Feldman, 2010a; Peltokorpi et al., 2015). This is because most employees are embedded in both their jobs and organizations (Peltokorpi et al., 2015). In organizational studies, organizational embeddedness refers to the sum of complex factors that prevent employees from leaving the organization (Yao et al., 2004). Organizational embeddedness consists of three interrelated dimensions: links, fit, and sacrifice (Mitchell et al., 2001). Employees become deeply rooted in the organization through formal and informal relationships, interactions within the organization, and comparing the benefits and costs associated with staying at it (Halbesleben and Wheeler, 2008; Lee et al., 2014). Previous studies found that compensation, psychological empowerment, and learning orientation are antecedents of organizational embeddedness (Bergiel et al., 2009; Karatepe, 2013; Tian et al., 2016; Van Dyk et al., 2013; Yoon et al., 2022). Furthermore, organizational embeddedness positively impacts organizational citizenship behavior, job performance, and organizational commitment, while reducing turnover and absenteeism (Lee et al., 2004; Lee et al., 2014; Mitchell et al., 2001; Yoon et al., 2022).

We argue that organizational embeddedness positively mediates the relationship between identity leadership and creative performance for the following reasons. Identity leadership fosters employees’ adoption of the organization’s values and identity by establishing a shared sense of identity. Employees with high organizational identification can develop links, a component of organizational embeddedness, which enhances cooperation and interaction (Dukerich et al., 2002). Employees’ organizational identity promotes loyalty and commitment, aiding in the internalization of the organization’s values and beliefs (Ashforth and Mael, 1989). Thus, employees strive to contribute to the organization. We therefore anticipate that identity leadership positively influences organizational embeddedness by internalizing employees’ organizational values and beliefs. Moreover, identity leadership, a relational leadership type, emphasizes the “we” to motivate employees, highlighting the interaction and relationship between employees and leaders. Social interactions within the organization strengthen bonds with leaders, fostering emotional attachment to the organization (Allen, 2006; Allen and Shanock, 2013). High levels of connectedness create a sense of obligation, which makes it difficult for individuals to leave the organization (Holtom and O’Neill, 2004; Kiazad et al., 2015). Thus, interactions through identity leadership are expected to enhance connectedness and obligation, positively influencing organizational embeddedness by making it more difficult for employees to leave. Job embeddedness is formed based on social connectedness within the organization. Employees’ sense of obligation enhances job performance (Ng and Feldman, 2010b). Employees with high organizational embeddedness have close connections within the organization, facilitating quick and easy innovation processes among employees (Ng and Feldman, 2010b). Similarly, organizational embeddedness helps achieve innovation. Thus, we anticipate that organizational embeddedness positively impacts creative performance through interactions and close exchanges among employees. Employees consider the sacrifices they would face upon leaving the organization, based on which they are likely to behave more innovatively and creatively to avoid potential sacrifices and strengthen their job security (Ng and Feldman, 2010b). Thus, organizational embeddedness encourages individuals to consider job security and relationships, increasing their motivation to contribute to the organization. Consequently, employees will enhance creative performance through engaging in new initiatives and creative processes. We therefore anticipate that identity leadership will positively influence organizational embeddedness and make it difficult for employees to leave the organization. In addition, organizational embeddedness is expected to positively impact employees’ creative performance because it stimulates their motivation. As such, we hypothesized:

*Hypothesis 3* (H3). Organizational embeddedness positively mediates the relationship between identity leadership and creative performance.

### 2.4 Integrated model: a serial mediating effect

Leadership plays a crucial role in cultivating organizational identification, which enhances employees’ efforts for the organization. Identity leadership promotes organizational identification by aligning employees’ identities with those of the organization through the notion of “we” (Steffens et al., 2014). We anticipate that identity leadership, through expressing, developing, creating, and internalizing social identities, will enable employees to derive relational energy within the organization, facilitating active interactions. Consequently, employees with relational energy build close relationships with peers and leaders, which fosters a sense of attraction to the organization. It is expected that this process positively affects organizational embeddedness. Thus, we anticipate serial processes of relational energy and organizational embeddedness in the relationship between identity leadership and creative performance.

Identity leadership demonstrates role modeling by embodying the leader’s identity prototypicality to employees (Hogg, 2001). Identity leadership operationalizes a shared vision, encouraging employees to embrace the organization’s identity (Halevy et al., 2011). When organizational vision is articulated, employees contribute to goal achievement and creative performance. Here, identity leadership defines and communicates what “we” signifies, creating a shared identity (Augoustinos and De Garis, 2012; Hogg and Giles, 2012; Seyranian and Bligh, 2008). Specifically, it cultivates a shared sense of “we,” uniting employees, and of cohesion by making all employees feel part of the same group (Steffens et al., 2014). This cohesion enhances relational energy through active interactions, which arise from social interactions within the organization (Baker, 2019; Owens et al., 2016). This process forms links that are a component of organizational embeddedness. Active social interactions facilitated by identity leadership sequentially increase relational energy and organizational embeddedness, positively influencing creative outcomes. Ultimately, identity leadership positively affects creative performance via relational energy and organizational embeddedness. Furthermore, it fosters close relationships within the organization based on a shared sense of “we” and elevates relational energy. It also ensures a high degree of fit between individuals and the organization by instilling similar characteristics, identities, and values, promoting organizational-personal congruence. This alignment of identity and characteristics occurs when employees share similar traits, goals, values, and cultures (Liu et al., 2010). Such organizational-personal fit can contribute to organizational commitment, which prevents employees from leaving the organization. Moreover, fit, a component of organizational embeddedness, enhances employees’ dedication and fosters positive attitudes toward their work; thus, it is expected to positively influence creative outcomes (Farzaneh et al., 2014).

In sum, identity leadership enhances cohesion within the organization, increasing relational energy. Relational energy increases employees’ attachment and desire to remain within the organization. Through these processes, organizational embeddedness is increased. Relational energy and organizational commitment can positively influence creative performance as they stimulate employees to generate constructive ideas and engage in proactive behaviors (Amah, 2016; Owens et al., 2016). Accordingly, we hypothesized:

*Hypothesis 4* (H4). Relational energy and organizational embeddedness will serially mediate the relationship between identity leadership and creative performance, such that identity leadership increases relational energy, which increases organizational embeddedness. This increased organizational embeddedness increases creative performance.

**Figure 1:**
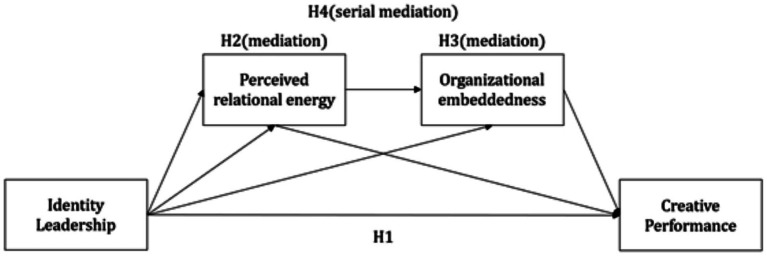
Hypothesized research model.

The hypothesized research model is presented in Figure 1.

## 3 Methodology

### 3.1 Sample and procedure

We collected data from manufacturing and service companies located in South Korea. Manufacturing sectors require creative outcomes, including useful ideas, technological development to survival (Cristofaro et al., 2022). This necessity is especially evident in South Korea, where manufacturing-centered industries predominate. Similarly, service sector facing competition and rapid market complexity are pursuing innovation by fostering employee creativity to secure competitive advantages (Tsai and Huang, 2020). Notably, service companies in Asia are assuming an increasingly critical role in economic development (Park and Shin, 2012). Therefore, service companies concentrate their efforts on strengthening employees’ creative and innovation capabilities (Melton and Hartline, 2013; Tsai and Huang, 2020). In this vein, in order to achieve the objective of this study which is the determinants of employees’ creative performance, we targets employees from manufacturing and service sectors in South Korea.

In our empirical analysis, companies with foreign ownership or investment in Korea were excluded from our sample because their organizational and cultural contexts differ from those of domestic firms. A unique characteristic distinguishing foreign ownership or investment companies from domestic firms is that ownership and control are held by non-Korean individuals whose national and cultural identities differ from those of managers in Korea (Lee et al., 2017).

We adopted a simple random sampling method to enhance sample representativeness and reduce sampling bias (Sekaran and Bougie, 2016; Zhang et al., 2025). We carried out data collection through the following procedures. First, we contacted HR managers at several companies which were randomly selected manufacturing and service sector across South Korea on a nationwide basis. We requested permission to conduct a survey of their employees. Second, we explained the purpose of the research and the anonymity assurance to the HR managers of each company through either on-site visits or online communication channels. Third, all participants received comprehensive information regarding the study’s objectives and survey process. Prior to data collection, informed consent was secured from each respondent, who was informed of their right to discontinue participation at any point without penalty. Subsequently, we distributed online or paper questionnaires to randomly selected employees within these companies. In total, 420 questionnaires were collected from the employees. The final analysis used data from 397 questionnaires after excluding incomplete responses and insincere responses.

Regarding the demographic characteristics of the sample, the average age of employees was 41.88 years (SD = 9.73), 59.9% were men, and the average employee tenure was 9.36 years (SD = 7.87). Education was measured by the total years of education including compulsory education. The average education was 15.59 years (SD = 2.04). Among the 397 respondents, 26.4% were staff, 22.9% were assistant managers, 29% were managers, 17.6% were senior managers, and 4% were executives. Last, 52.4% were employed in office jobs, 15.1% in technical jobs, 17.1% in service or sales jobs, 10.8% in R&D jobs, and 4.5% in other types of jobs.

### 3.2 Measures

The questionnaires in this study were originally prepared in English (see Appendix A). Therefore, the traditional method of back translation (Brislin, 1980) was used to translate the English language questionnaires into Korean. All questions were measured on a five-point Likert scale. The operational definitions of key variables are shown in Table 1.

**Table 1:** Operational definition of key variables.

Variable	Operational definition	References
Identity leadership	Identity leadership is represented by the degree to which leader was seen to act as identity leader using the four dimensions (identity prototypicality, identity advancement, identity entrepreneurship, and identity impresarioship) of the leader’s shared sense of “us” and “we”	Steffens et al. (2014) and Van Dick et al. (2018)
Relational energy	Relational energy captures the degree to which organizational relationships generate energy by creating feelings of positive engagement, vitality, and eagerness to act.	Boyatzis and Rochford (2020) and Owens et al. (2016)
Organizational embeddedness	Organizational embeddedness is a force that binds the individual to the organization. Organizational embeddedness rated the links and fit between the organization and employee, and the degree of sacrifice incurred upon leaving the organization.	Crossley et al. (2007), Lee et al. (2022) and Mitchell et al. (2001)
Creative performance	Creative performance is operationally defined by three dimensions measuring the degree to which an individual generates, promotes, and realizes new and creative ideas at work.	George and Zhou (2002), Shalley et al. (2009) and Zhang et al. (2021)

#### 3.2.1 Identity leadership

Identity leadership define as a leader’s capability and process to express, develop, create, and internalize a shared social identity for employees based on a shared feeling of “us” (Steffens et al., 2014). Identity leadership was measured using 15 items from Steffens et al. (2014). The items included the following: “My leader is a model member of the group” and “My leader devises activities that bring the group together.” Cronbach’s alpha was 0.962.

#### 3.2.2 Relational energy

Relational energy can be understood as the energizing effect generated through workplace interactions, which serves to elevate one’s performance capacity (Owens et al., 2016). Relational energy was measured using five items from Owens et al. (2016). The items included the following: “After interacting with my immediate supervisor, I feel more energy to do my work” and “I feel increased vitality when I interact with my immediate supervisor.” Cronbach’s alpha was 0.930.

#### 3.2.3 Organizational embeddedness

Organizational embeddedness refers to the sum of complex factors that prevent employees from leaving the organization (Yao et al., 2004). Organizational embeddedness was measured using seven items from Crossley et al. (2007). The items included the following: “I’m too caught up in this organization to leave” and “I feel tied to this organization.” Cronbach’s alpha was 0.830.

#### 3.2.4 Creative performance

Creative performance is defined as employees’ ability to produce novel and practical outcomes, such as products, processes, or services in organizations (Woodman et al., 1993). Creative performance was measured using 13 items from George and Zhou (2002). The items included the following: “I suggest new ways of performing work tasks” and “I come up with creative solutions to problems.” Cronbach’s alpha was 0.937.

## 4 Results

### 4.1 Correlation and reliability analysis

Table 2 reports the descriptive statistics and correlations among the key variables. As expected, there was a positive correlation between creative performance and organizational embeddedness (*r* = 0.393, *p* < 0.001), creative performance and relational energy (*r* = 0.466, *p* < 0.001), and creative performance and identity leadership (*r* = 0.470, *p* < 0.001). Moreover, our key variables demonstrated an acceptable degree of internal consistency.

**Table 2:** Descriptive statistics and correlations.

Variable		Mean	SD	1	2	3	4	5	6	7	8	9	10
1	Gender	0.40	0.49										
2	Age	41.88	9.73	−0.337***									
3	Tenure	9.36	7.87	−0.283***	0.490***								
4	Position	2.50	1.17	−0.361***	0.487***	0.499***							
5	Education	15.59	2.04	−0.133**	0.093	0.174***	0.350***						
6	Job type	2.00	1.24	−0.116*	0.014	−0.014	0.029	0.188***					
7	Identity leadership	3.31	0.83	−0.080	0.111*	0.115*	0.086	0.075	−0.038	(0.962)			
8	Relational energy	2.86	0.95	−0.114*	0.128*	0.094	0.110*	0.032	−0.025	0.772***	(0.930)		
9	Organizational embeddedness	3.01	0.85	−0.149**	0.259***	0.210***	0.207***	0.064	−0.072	0.426***	0.461***	(0.830)	
10	Creative performance	3.33	0.68	−0.220***	0.177***	0.194***	0.306***	0.225***	0.077	0.470***	0.466***	0.393***	(0.937)

*N* = 397. *p < 0.05; **p < 0.01, ***p < 0.001; Cronbach’s alpha values are reported in parentheses on the diagonals.

### 4.2 Confirmatory factor analysis

We conducted a confirmatory factor analysis (CFA) to test the construct validity of study variables. As Table 3 shows, the four-factor model fit the data reasonably well (*χ*^2^(df) = 1098.166(554), CFI = 0.949, TLI = 0.945, RMSEA = 0.050). All the CFA indicators satisfied the cutoff; thus, we considered our hypothesized measurement model appropriate for the data. Moreover, the average variances extracted (AVE) and composite reliability (CR) values for all variables satisfied the criteria (Fornell and Larcker, 1981). Because all data of the key variables were self-reported, common method bias is a possibility. Thus, following the recommendation of Podsakoff et al. (2003), Harman’s one-factor test was used to test for the presence thereof. Our results showed that the first factor was 43.03%. Therefore, we judged that the probability of common method bias was low (Podsakoff et al., 2003).

**Table 3:** Model fit statistics for measurement models.

Measurement model	*χ*^2^ (df)	CFI	TLI	RMSEA	Δ*χ*^2^ (df)
Theoretical 4-factor model (IL, RE, OE, CP)	1098.166 (554)	0.949	0.945	0.050	
3-Factor model (IL, RE & OE merged, CP)	1541.961 (557)	0.907	0.901	0.067	443.795 (3)^***^
2-Factor model (IL & RE & OE merged, CP)	2072.437 (559)	0.857	0.848	0.083	974.271 (5)^***^
1-Factor model	4043.011 (560)	0.671	0.651	0.125	2944.845 (6)^***^

IL, Identity leadership; RE, Relational energy; OE, Organizational embeddedness; CP, Creative performance; *χ*^2^, Chi-square; *df*, degrees of freedom; RMSEA, root mean square error of approximation; CFI, comparative fit index; TLI, Tucker-Lewis index; ***p < 0.001.

### 4.3 Hypothesis testing

We used hierarchical regression analysis and Hayes (2013) Process Macro to test the hypothesized relationships. Table 4 provides the results of the test. H1 predicted that identity leadership would have a positive effect on creative performance. In Model 6, identity leadership was found to positively affect creative performance (*β* = 0.441, *p* < 0.001), supporting H1.

**Table 4:** Results of regression analyses and bootstrapped indirect effects test.

Variables	Relational energy		Organizational embeddedness		Creative performance				
	Model 1	Model 2	Model 3	Model 4	Model 5	Model 6	Model 7	Model 8	Model 9
Gender	−0.073	−0.041	−0.054	−0.038	−0.114*	−0.095*	−0.085	−0.087	−0.081
Age	0.078	0.026	0.174**	0.147**	0.014	−0.016	−0.022	−0.045	−0.046
Tenure	0.016	−0.034	0.075	0.049	0.036	0.008	0.016	−0.002	0.006
Position	0.037	0.049	0.060	0.067	0.194**	0.202***	0.190**	0.188**	0.181**
Education	0.006	−0.046	0.023	−0.004	0.128*	0.099*	0.109*	0.099*	0.108*
Job type	−0.037	0.006	−0.086	−0.064	0.034	0.059	0.057	0.071	0.068
Identity leadership		0.770***		0.393***		0.441***	0.262***	0.363***	0.235***
Relational energy							0.232***		0.181**
Organizational embeddedness								0.197***	0.168***
*R* ^2^	0.025	0.603	0.089	0.240	0.125	0.315	0.336	0.345	0.357
Δ*R*^2^		0.578***		0.151***		0.190***	0.021***	0.030***	0.012**

Indirect effects	Estimate	Boot SE	Lower level	Upper level
IL → RE → CP	0.1140	0.0473	0.0209	0.2056
IL → OE → CP	0.0219	0.0131	0.0008	0.0516
IL → RE → OE → CP	0.0322	0.0133	0.0102	0.0620

*N* = 397; IL, Identity leadership; RE, Relational energy; OE, Organizational embeddedness; CP, Creative performance; Standardized regression coefficients reported; The indirect effects estimate was tested for significance using 95% bias-corrected bootstrapped confidence intervals (10,000 times); *p < 0.05, **p < 0.01, ***p < 0.001 (two-tailed).

To test the mediating role of relational energy, we followed the procedure of Baron and Kenny (1986). First, for H1, we tested whether identity leadership is positively related to creative performance. Second, we found that identity leadership was positively associated with relational energy (*β* = 0.770, *p* < 0.001) in Model 2. Third, relational energy was positively related to creative performance (*β* = 0.232, *p* < 0.001) in Model 7, and significant additional variance was explained therein (Δ*R*^2^ = 0.021, *p* < 0.001). The effect of identity leadership on creative performance weakened, but was still significant (*β* = 0.262, *p* < 0.001), suggesting partial mediation. However, this indirect method has a possibility of statistical errors (Hayes and Scharkow, 2013). Therefore, we additionally conducted the bootstrap method for more reliable estimates (Preacher and Hayes, 2004; Shrout and Bolger, 2002). The results indicate that the mediating role of relational energy was significant (indirect effect = 0.1140, Boot SE = 0.0473, 95%CI[0.0209, 0.2056]). Thus, H2 was supported.

To test the mediating role of organizational embeddedness, we again followed Baron and Kenny (1986). First, for H1, we tested whether identity leadership is positively related to creative performance. Second, we found that identity leadership was positively associated with organizational embeddedness (*β* = 0.393, *p* < 0.001) in Model 4. Third, organizational embeddedness was positively related to creative performance (*β* = 0.197, *p* < 0.001) in Model 8, and significant additional variance was explained therein (Δ*R*^2^ = 0.030, *p* < 0.001). The effect of identity leadership on creative performance weakened, but was still significant (*β* = 0.363, *p* < 0.001), suggesting partial mediation. We additionally conducted the bootstrap method for more reliable estimates (Preacher and Hayes, 2004; Shrout and Bolger, 2002). The results indicate that the mediating role of organizational embeddedness was significant (indirect effect = 0.0219, Boot SE = 0.0131, 95%CI [0.0008, 0.0516]). Thus, H3 was supported.

H4 predicted that relational energy and organizational embeddedness would serially mediate the relationship between identity leadership and creative performance. We used Hayes (2013) Process Macro Model 6 to test H4. The indirect test with 10,000 bootstrapped samples produced a coefficient of 0.0322 and the 95% CI excluded zero [0.0102, 0.0620]. Thus, our results support H4.

## 5 Conclusion and implications

Leadership is an important antecedent for achieving organizational goals and survival, and research has explored various leadership styles to enhance organizational performance and innovation (Inceoglu et al., 2018; Hughes et al., 2018). To address the gap in previous studies, this one aimed to elucidate the effects of identity leadership. Specifically, we hypothesized a positive relationship between identity leadership and employees’ creative performance. Furthermore, this study sought to clarify the mediating and serial mediating roles of relational energy and organizational embeddedness in the relationship between identity leadership and creative performance. The results are as follows. First, our results indicated that identity leadership positively affects creative performance. Second, relational energy positively mediates the relationship between identity leadership and creative performance. Third, organizational embeddedness positively mediates the relationship between identity leadership and creative performance. Last, relational energy and organizational embeddedness serially mediate the relationship between identity leadership and creative performance.

Our findings highlight the following theoretical implications. First, this study deepens understanding of identity leadership by testing the role of identity leadership in employees’ creative performance. Previous studies focused on the effects of identity leadership after developing identity leadership measurements and suggesting identity leadership-specific behaviors (Krug et al., 2021; Fransen et al., 2020; Van Dick et al., 2018). However, research is lacking on how identity leadership impacts employees’ creative performance, which is fundamental to organizational innovation. Thus, this study extends prior research by focusing on the positive role of identity leadership in creative performance.

Second, we found that identity leadership is effective in collectivist cultures such as that in South Korea. East Asian cultures value attending to others, fitting-in, and harmonious interactions (Bagozzi and Lee, 2002). Koreans tend to see social relationships as extensions of family relationships, and these are based on a sense of “we-ness.” This also cultivates strong connectedness with group members at work (Wilderom et al., 2015). As the concept of “we” is important in South Korea, empirical evidence shows that identity leadership is effective. Our findings are consistent with those of prior identity leadership research tested in the Western context. Through the results of this study, we have expanded understanding of the effectiveness of identity leadership.

Third, research is lacking on the role of relational energy in organizational studies. Previous studies showed that relational energy positively affects job engagement and job performance (Owens et al., 2016). Our results highlight the mediating role of relational energy between identity leadership and creative performance. Thus, this study contributes to leadership and organizational research.

Fourth, previous research on organizational embeddedness focused on its relationship with leader-member exchange, organizational citizenship behavior, job performance, and turnover intention (Harris et al., 2011; Lee et al., 2004; Peltokorpi et al., 2015). While some studies suggested that organizational embeddedness positively impacts innovation (Ng and Feldman, 2010b), research on the relationship between leadership, organizational embeddedness, and creativity is limited. This study found that organizational embeddedness is an antecedent that enhances creative performance. Thus, it provides theoretical implications by identifying the role of organizational embeddedness in the relationship between identity leadership and creative performance.

Fifth, we applied a serial mediation model to test the impact and process of identity leadership on creative performance. Specifically, this study highlights the roles of relational energy and organizational embeddedness as antecedents for enhancing creative performance. While the components of organizational embeddedness include links, fit, and sacrifice (Felps et al., 2009), prior studies also identified organizational support and growth opportunities as determinants thereof (Nguyen et al., 2017). Our results indicate that relational energy obtained from organizational leaders prevents employees from leaving. As such, this study reveals the serial effects of identity leadership on relational energy and organizational embeddedness to enhance creative performance. Thus, this study provides theoretical contributions to leadership and organizational behavior research.

Lastly, by drawing on social cognition theory as the overarching theoretical framework, we contribute to the fields of creativity and leadership studies by uncovering a mechanism that explains the relationship between identity leadership and employees’ creative performance through relational energy and organizational embeddedness. Korean Confucianism and collectivist culture emphasize harmony than individuals’ preference within organization (Lee et al., 2018; Sung and Choi, 2021). In this context, our findings extended the applicability of social cognitive theory to leadership research by demonstrating that leaders who promote a sense of “us” identity shape employees’ cognition of relational value and organizational importance, which sequentially enhances their creative performance in the Korean context.

The results of this study also have practical implications. First, this study highlights identity leadership as important for enhancing creative performance. In particular, Korea’s collectivist culture requires a leadership style that emphasizes the “we.” Therefore, systematic training programs and top management’s attention are needed to promote identity leadership at the organizational level. Organizations should design identity leadership enhancement programs for top executives and middle managers. For example, leadership training and education programs at organizational level can enhance manager’s identity leadership related capabilities which in turn foster a sense of “we” among employees and creative self-efficacy to promote creative performance.

Second, to strengthen a shared sense of “We” through Collective Identity Building, Managers should actively cultivate a strong sense of shared organizational identity. This can be achieved by clearly communicating the organization’s vision, values, and collective goals. Managers also should encourage for employees to involve in decision-making and goal-setting processes to make them feel part of the “ingroup within organization.”

Finally, our results also indicate the need for relational energy as an antecedent to fostering creative performance. Organizations should establish support systems to help employees gain relational energy through interactions with leaders and colleagues. Moreover, a horizontal organizational structure should be designed to facilitate communication.

Despite the theoretical and practical implications, this study has several limitations. First, it applied a serial mediation model to explore the relationship between identity leadership and creative performance. However, the effect of leadership on performance can differ depending on situational factors. Therefore, future research should consider various moderating variables to analyze the effects of identity leadership. Second, data were collected via a self-reported questionnaire, which may present common method bias. To minimize bias, future research should separate the sources of measurement for independent and dependent variables. Third, this study used cross-sectional data, although the effects of leadership can change over time. Therefore, future research should apply a longitudinal design to enhance reliability. Fourth, we performed an individual-level analysis to explain factors affecting creative performance. However, leadership research requires team-level research. Therefore, our findings can be extended by examining the effects of team-level factors based on multi-level analyses in future studies. Last, we collected survey data for employees in South Korea. We are uncertain about the extent to which our findings are generalizable to other cultural contexts. Future research that replicates this study in a cross-cultural context, preferably using data from other cultural contexts, would enhance the ability to generalize our findings.

## Data Availability

The raw data supporting the conclusions of this article will be made available by the authors, without undue reservation.

## Appendix

Appendix A. The questionnaire items.

**Identity leadership** (α= 0.962) (Steffens et al., 2014)

Identity prototypicality: ‘Being one of us’

My leader embodies what the group stands for.

My leader is representative of members of the group.

My leader is a model member of the group.

My leader exemplifies what it means to be a member of the group.

Identity advancement: ‘Doing it for us’

My leader promotes the interests of members of the group.

My leader acts as a champion for the group.

My leader stands up for the group.

When my leader acts, he or she has the group interests at heart.

Identity entrepreneurship: ‘Crafting a sense of us’

My leader makes people feel as if they are part of the same group.

My leader creates a sense of cohesion within the group.

My leader develops an understanding of what it means to be a member of the group.

My leader shapes members' perceptions of the group values and ideals.

Identity impresarioship: ‘Making us matter’

My leader devises activities that bring the group together.

My leader arranges events that help the group function effectively.

My leader creates structures that are useful for the group.

**Relational energy** (α= 0.930) (Owens et al., 2016)

I feel invigorated when I interact with immediate supervisor.

After interacting with immediate supervisor I feel more energy to do my work.

I feel increased vitality when I interact with immediate supervisor.

I would go to immediate supervisor when I need to be “pepped up”.

After an exchange with immediate supervisor, I feel more stamina to do my work.

**Organizational embeddedness** (α= 0.830) (Crossley et al., 2007)

I feel attached to this organization.

It would be difficult for me to leave this organization.

I’m too caught up in this organization to leave.

I feel tied to this organization.

I simply could not leave the organization that I work for.

It would be easy for me to leave this organization. (R)

I am tightly connected to this organization.

**Creative performance** (α= 0.937) (George and Zhou, 2002).

I suggest new ways to achieve goals or objectives.

I come up with new and practical ideas to improve performance.

I search out new technologies, processes, techniques, and/or product ideas.

I suggest new ways to increase quality.

My thoughts are a good source of creative ideas.

I am not afraid to take risks.

I promote and champion ideas to others.

I exhibit creativity on the job when given the opportunity to.

I develop adequate plans and schedules for the implementation of new ideas.

I often have new and innovative ideas.

I come up with creative solutions to problems.

I often have a fresh approach to problems.

I suggest new ways of performing work tasks.
